# P‐TEFb goes viral

**DOI:** 10.1002/icl3.1037

**Published:** 2015-11-25

**Authors:** Justyna Zaborowska, Nur F. Isa, Shona Murphy

**Affiliations:** ^1^ Sir William Dunn School of PathologyUniversity of OxfordOxfordUK; ^2^ Department of BiotechnologyKulliyyah of Science, IIUMKuantanPahangMalaysia

**Keywords:** CDK9, CTD, EBV, HIV, HSV, HTLV, P‐TEFb

## Abstract

Positive transcription elongation factor b (P‐TEFb), which comprises cyclin‐dependent kinase 9 (CDK9) kinase and cyclin T subunits, is an essential kinase complex in human cells. Phosphorylation of the negative elongation factors by P‐TEFb is required for productive elongation of transcription of protein‐coding genes by RNA polymerase II (pol II). In addition, P‐TEFb‐mediated phosphorylation of the carboxyl‐terminal domain (CTD) of the largest subunit of pol II mediates the recruitment of transcription and RNA processing factors during the transcription cycle. CDK9 also phosphorylates p53, a tumor suppressor that plays a central role in cellular responses to a range of stress factors. Many viral factors affect transcription by recruiting or modulating the activity of CDK9. In this review, we will focus on how the function of CDK9 is regulated by viral gene products. The central role of CDK9 in viral life cycles suggests that drugs targeting the interaction between viral products and P‐TEFb could be effective anti‐viral agents.

## Introduction

Transcription of many human viruses is dependent on host cell factors such as RNA polymerase II (pol II) 1. Pol II is made up of 12 subunits (Rpb1–Rpb12) 2. The carboxyl‐terminal domain (CTD) of the largest subunit of pol II (Rpb1) comprises tandemly repeated heptapeptides with the consensus sequence Tyr1‐Ser2‐Pro3‐Thr4‐Ser5‐Pro6‐Ser7 3, 4. The CTD plays a central role in transcriptional and co‐transcriptional RNA processing 4, 5, 6, 7 by mediating the recruitment of transcription and processing factors at different steps of the transcription cycle through reversible modification of the residues within the heptapeptide repeats 8. CTD modification generates a code that regulates the interaction with transcription and RNA processing factors 9, 10, 11, 12. Among the CTD modifications, the phosphorylation of Ser2 (Ser2P) of pol II CTD, is catalyzed by the cyclin‐dependent kinase 9 (CDK9) subunit of the positive transcription elongation factor b (P‐TEFb). P‐TEFb also phosphorylates two negative transcription elongation factors: DRB sensitivity‐inducing factor (DSIF) and negative elongation factor (NELF), which are recruited to the elongation complex in the promoter proximal region to induce pol II pausing 13, 14. Phosphorylation of NELF promotes its dissociation from the elongation complex, whereas phospho‐DSIF remains associated with pol II as an elongation activator 14. The role of P‐TEFb in stimulating productive elongation was elucidated using 5,6‐dichloro‐1‐β‐d‐ribofuranosylbenzimidazole (DRB), which is a purine nucleoside analog that inhibits CDK9 activity 15. Treatment of cells with DRB results in the production of shortened transcripts, diagnostic of the inability of pol II to transcribe through the early elongation checkpoint in the absence of P‐TEFb activity 16. In addition to Ser2 of the pol II CTD, NELF and DSIF, several serine residues in the key transcription factor p53 are phosphorylated by CDK9, which results in p53 activation 17, 18.

**Figure 1:**
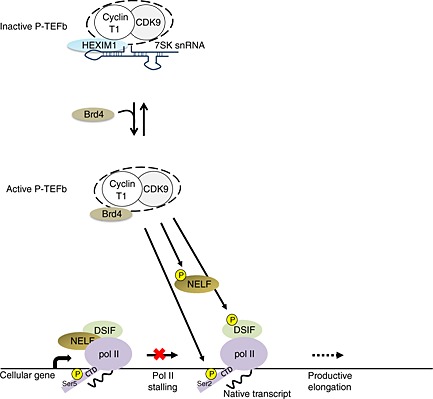
The role of positive transcription elongation factor b (P‐TEFb) in a regulation of pol II transcription. P‐TEFb, comprising cyclin‐dependent kinase 9 (CDK9) and a cyclin T1 subunit, exists in a catalytically inactive small nuclear ribonucleic particle with 7SK snRNA and HEXIM1. Active P‐TEFb is bound instead to bromodomain‐containing protein 4 (Brd4) and stimulates elongation through phosphorylation of the pol II carboxyl‐terminal domain (CTD) on Ser2 and two negative transcription elongation factors: DRB sensitivity‐inducing factor (DSIF) and negative elongation factor (NELF). Phosphorylation converts DSIF from a repressor to an activator, whereas phosphorylated NELF leaves the elongation complex.

Positive transcription elongation factor b comprises the CDK9 kinase and a cyclin partner 15. CDK9 is an ubiquitously expressed Ser/Thr proline‐directed kinase 19, 20. It was identified in the early 1990s and initially named PITALRE 19, 21. It exists in two isoforms, CDK9‐42 and CDK9‐55, which arise from two transcription start sites 22. The ratio of CDK9‐42/CDK9‐55 is cell type‐dependent 23, and it is unclear if there are functional differences between the two isoforms. CDK9 interacts with T‐type cyclins – T1, T2a, T2b and cyclin K 24, 25. In this heterodimer, the CDK9 subunit provides the enzymatic activity, while the cyclin has a regulatory role 24, 26. However, mass spectrometry has failed to demonstrate that cyclin K is associated with CDK9 27. In addition, a recent study revealed that cyclin K associates with CDK12 and CDK13 28. Therefore, cyclins T1 and T2 may be the major cyclins associated with CDK9. In the cell, P‐TEFb exists in two major forms. A large amount of cellular P‐TEFb is sequestered in an inactive complex with the noncoding 7SK small nuclear RNA (7SK snRNA), hexamethylene bisacetamide‐induced protein 1 (HEXIM1) and/or HEXIM2 and the La‐related protein 7 29, 30, 31, 32, 33, 34, 35. The catalytically active form of P‐TEFb is instead generally associated with bromodomain‐containing protein 4 (Brd4) and the super elongation complex (SEC) 36, 37, 38. The transition between active and inactive forms is dynamic and tightly regulated 37, 38, reflecting the important role that P‐TEFb plays in cellular processes (Fig. 1). Given the importance of P‐TEFb in regulating cellular gene expression, it is perhaps not surprising that this complex is also functionally integrated into the expression of human viruses (Table 1). Several viral factors regulate CTD phosphorylation through recruitment or modulation of the activity of CDK9.

**Table 1:** Viral factors that interact with P‐TEFb

Virus	Viral factors that interact with P‐TEFb	References
Herpes simplex virus	ICP22	50, 142
Human cytomegalovirus	pUL69	69, 70, 143
	IE2 86	
	pUL97	
Epstein–Barr virus	EBNA2	75
Human immunodeficiency virus	Tat	144, 145
Human T‐lymphotropic virus	Tax	110, 111
Human adenovirus	E1A	117
Influenza A virus	vRNP	121
Dengue virus	DENV core protein	129
Kaposi's sarcoma‐associated virus	K‐cyclin	59

P‐TEFb, positive transcription elongation factor b; ICP22, immediate‐early protein 22; EBNA2, Epstein–Barr nuclear antigen 2; vRNP, viral RNA‐dependent RNA polymerases.

The studies reviewed here present examples of DNA and RNA viruses that subvert the host cell CTD kinase CDK9 for their own needs. Understanding the requirements of CDK9 for viral infection and how viral infection alters pol II CTD phosphorylation patterns not only advances our knowledge of viral pathogenesis but also provides potential new anti‐viral drug targets.

### The α‐herpesvirus ICP22 protein has an intimate relationship with P‐TEFb

**Figure 2:**
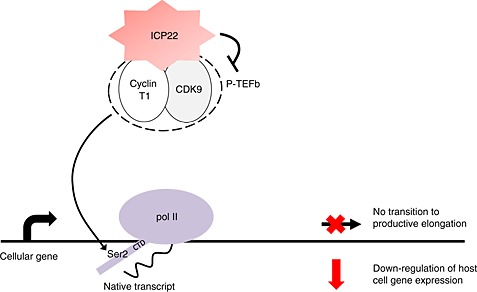
Immediate‐early protein 22 (ICP22) inhibits CDK9. HSV ICP22 interacts with cyclin‐dependent kinase 9 (CDK9). This results in the loss of Ser2 phosphorylation of the carboxyl‐terminal domain (CTD) of pol II and inhibition of transcription elongation of host cell genes. P‐TEFb, positive transcription elongation factor b.

After infection, herpes simplex virus (HSV‐1 and HSV‐2) goes through a lytic replicative phase and causes localized lesions. This is followed by a latent phase where viruses reside in sensory neurons and can be frequently reactivated by a range of stresses, including high temperature and ultraviolet light 39. Although HSV infections are generally self‐limiting in healthy individuals, they can cause cancer, inflammation of the brain and the eye, and pose a significant mortality risk in infants and immuno‐compromised adults 40, 41. The HSV immediate‐early protein 22 (ICP22) is encoded by the U_S_1 gene and comprises 420 amino acid residues. Viral deletion mutants without the U_S_1 gene have a reduced capacity to establish latency in animal models 42, 43. In addition, deletion of this gene causes viral growth defects in some cell lines 42. During HSV‐1 productive infection, three classes of genes are sequentially expressed; first, the immediate‐early genes (α‐genes), next, the early genes (β‐genes), and finally, the late genes (γ‐genes) 44, 45. It has been demonstrated that ICP22 represses transcription of the viral α‐genes, β‐genes and γ‐genes by blocking the recruitment of P‐TEFb to their promoters. In contrast, the herpes viral protein 16 (VP16) overcomes the inhibitory effects of ICP22 on α‐gene transcription 46. It has been demonstrated that ICP22 alters the CTD phosphorylation state of pol II 47. ICP22 mediates two distinct effects on pol II: the induction of an intermediately migrating form of pol II (pol IIi) and the loss of pol II forms phosphorylated on Ser2 47, 48. Pol IIi replaces the normal hyperphosphorylated form of pol II in HSV‐1 infected cells. The induction of pol IIi requires ICP22 and another viral factor, UL13 49. A recent study demonstrated that ICP22 interacts directly with cellular CDK9 and that a short segment of ICP22 (residue 193‐256) is sufficient for this interaction 50. However, this short segment does not interact with pol II, unlike the full length of ICP22. Nevertheless, ectopic expression of either full length of ICP22 or amino acids residues 193‐256, leads to the loss of Ser2 CTD phosphorylation and subsequently to the inhibition of transcriptional elongation of host cell genes 50. These findings suggest a model where ICP22 is recruited to the transcriptionally active host genes soon after infection, where its interaction with P‐TEFb inhibits the kinase activity of CDK9, resulting in the loss of productive elongation (Fig. 2). Lytic infection with HSV‐1 also causes a transcription termination defects on host cell genes 51. Termination of transcription of the viral genes is however, efficient, highlighting that HSV‐1 proteins regulate transcription to maximize expression of the viral genome while down‐regulating expression of the host genome. Shutting down host cell gene expression could benefit the virus by, for example, helping to evade anti‐viral responses including production of type I interferon 52. Interaction of ICP22 with the CDK9 target, p53, is also important for efficient HSV‐1 replication 53.

### KSHV factors regulate P‐TEFb activity towards pol II and p53

**Figure 3:**
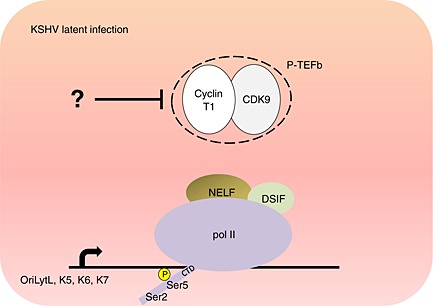
Pol II transcription complexes are paused at the promoters of Kaposi sarcoma‐associated herpesvirus (KSHV) lytic genes during latent infection. The action of negative elongation factor (NELF) and inhibition of cyclin‐dependent kinase 9 (CDK9) prevents expression of KSHV lytic genes OriLytL, K5, K6, and K7 during latency. Inhibition of CDK9 prevents phosphorylation of Ser2 of the pol II carboxyl‐terminal domain (CTD), whereas Ser5 is hyperphosphorylated.

Kaposi sarcoma‐associated herpesvirus (KSHV) is also known as human herpesvirus 8. KSHV causes Kaposi's sarcoma, multicentric Castleman's disease, and primary effusion lymphoma 54, 55, 56. K‐cyclin, encoded by the virus, can interact and activate CDK6 and can trigger apoptosis in cells with high levels of this cyclin 57, 58. In the search for new K‐cyclin partners, CDK9 was identified as a new interacting CDK. In addition, it was demonstrated that K‐cyclin stimulates CDK9 kinase activity towards p53, and that CDK9 is required for K‐cyclin‐induced p53‐dependent growth suppression 59. However, the role of CDK9 in KSHV replication goes beyond its interaction with K‐cyclin. The replication and transcription activator (RTA) of KSHV, K‐RTA, regulates the reactivation of KSHV from a latent state. As demonstrated by an *in vitro* kinase assay, RTA is a substrate of CDK9. Furthermore, CDK9 inhibitors suppress the expression of various K‐RTA target genes. This further suggests that CDK9 inhibitors could be used to disturb KSHV replication 60. Interestingly, transcription elongation of KSHV lytic genes is paused during latency and can be reactivated in an RTA‐independent manner. Chip‐on‐chip analysis revealed that during KSHV latent infection, pol II transcription complexes are associated with NELF at the promoters of a group of lytic genes including OriLytL, K5, K6, and K7 (Fig. 3). The CTD of pol II at these promoters is hyperphosphorylated on Ser5 and hypophosphorylated of Ser2. It was hypothesized that hypophosphorylation of Ser2 might be due to the recruitment of enzymatically inactive P‐TEFb to promoters 61. Therefore, the negative control of transcriptional elongation of lytic gene expression by modulating CDK9 activity during KSHV latency could be a key regulatory mechanism.

### P‐TEFb is a key player in HCMV trancriptosomes

The β‐herpesvirus human cytomegalovirus (HCMV), also known as human herpesvirus 5, infects 50–90% of the human population 62. HCMV infections are the leading viral cause of birth defects and pose a significant mortality risk in immunosuppressed individuals. In addition, HCMV may contribute to atherosclerosis and restenosis after coronary atherectomy 62, 63. HCMV infection results in the formation of viral transcriptosomes in the nucleus 64, 65, 66. These are the sites of transcription of the viral immediate‐early (IE) genes to which various viral and cellular factors are recruited 67. Cellular transcription regulators present at these sites include pol II, CDK9, cyclin T1, CDK7, and Brd4 65, 66, 68. It is well documented that HCMV infection affects the level and kinase activity of the recruited CDKs 68. In addition, recruitment of CDK9 to transcriptosomes during lytic infection results in hyperphosphorylation of the pol II CTD 66, 68, 69. Increased CTD phosphorylation might provide docking sites for processing factors regulating the alternative splicing of the primary HCMV transcripts 68. In addition, P‐TEFb activity is associated with the regulatory functions of HCMV‐encoded proteins pUL69, IE2‐86, and pUL97 67, 69, 70 and thus appears to be important for HCMV replication.

### P‐TEFb is critical for immortalization of EBV‐infected cells

The Epstein–Barr virus (EBV), a γ‐herpesvirus, is another important human pathogen, and seropositivity to EBV is estimated to be >90% in adults 71. EBV is able to infect and immortalize human B‐cells and is the causative agent of infectious mononucleosis 72, 73. It has been also associated with several cancers, including Burkitt lymphoma and subsets of Hodgkin's lymphomas and T‐cell lymphomas 74. One of the viral genes translated during latency and required for B‐cell transformation and proliferation of infected cells is Epstein–Barr nuclear antigen 2 (EBNA2) 75. EBNA2 is an activator of viral and cellular transcription, and P‐TEFb is required for its activity 76, 77, 78. EBNA2 activation is sensitive to inhibition by a dominant negative mutant of CDK9 and the CDK9 inhibitor DRB. In addition, EBNA2 promotes Ser5 phosphorylation of the pol II CTD 78. EBNA2 activates transcription from the viral C promoter (Cp) generating a long primary transcript encoding nuclear antigens necessary for immortalization of the host cells. In addition, EBNA2 activates the promoters of viral latent membrane protein genes (LMP1, LMP2A, and LMP2B) 79, 80, 81. Recently, Palermo *et al.* (2011) demonstrated that Cp directs the buildup of a high level of stalled pol II together with the pausing factors DSIF and NELF. The stalled pol II maintains a nucleosome‐depleted region as measured by chromatin immunoprecipitation for histone H3. Moreover, stalled pol II increases recruitment of Brd4‐associated P‐TEFb to drive high levels of Ser2 phosphorylation of the pol II CTD and facilitate productive elongation 82. These results highlight a key role for P‐TEFb in EBNA2‐dependent transcription activation and the immortalization of EBV‐infected cells.

### HIV Tat effectively hijacks P‐TEFb

The human immunodeficiency virus (HIV) is the causative agent of acquired immunodeficiency syndrome 83. The main target cells for HIV are CD4+ T lymphocytes and macrophages 84, 85. The most extensively studied *in vivo* latent reservoir is found within memory CD4+ T cells 86. HIV can also infect dendritic cells 87. Activation of latent viruses occurs at the level of elongation of transcription and requires binding of the HIV‐encoded elongation factor (Tat) to the transactivation response element at the start of HIV transcripts. The CDK9/cyclin T1 complex is an important factor for productive elongation of transcription of HIV genomes, and the inhibition of CDK9 can ameliorate HIV‐induced disease in animal models 88. In cultured human cells, dominant negative CDK9 mutants impair HIV‐1 replication 89.

**Figure 4:**
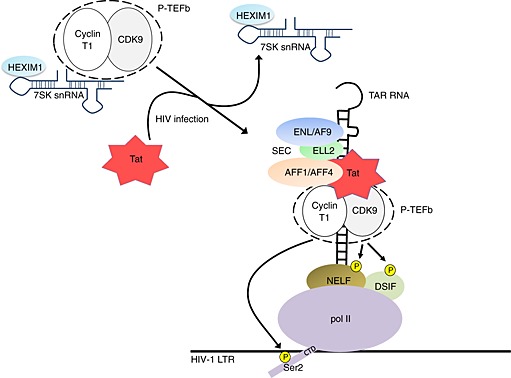
Human immunodeficiency virus type 1 (HIV‐1) Transactivator of transcription (Tat) stimulates transcription elongation by recruiting positive transcription elongation factor b (P‐TEFb). The HIV‐encoded Tat extracts the P‐TEFb from the inactive complex containing the 7SK small nuclear ribonucleic particle (7SK snRNP), targets cyclin‐dependent kinase 9 (CDK9) and a cyclin T1 to the transactivation response element (TAR) and assembles the super elongation complex (SEC) to activate HIV transcription. P‐TEFb triggers productive elongation by phosphorylating negative elongation factor (NELF), DRB sensitivity‐inducing factor (DSIF), and Ser2 of carboxyl‐terminal domain (CTD) of pol II. LTR, long terminal repeat.

Multiple levels of regulation of P‐TEFb impact HIV transcription. For example, P‐TEFb activity can be regulated by phosphorylation/dephosphorylation of the CDK9 subunit 90, and phosphatases involved in this process have been implicated in regulating HIV transcription 91, 92. HIV Tat targets CDK9/cyclin T1 to transactivation response element to stimulate transcription elongation through P‐TEFb‐dependent phosphorylation of NELF, DSIF, and Ser2 of pol II CTD 93, 94, 95. Tat competes with HEXIM1 for binding to cyclin T1 to promote the dissociation of P‐TEFb from the large inactive complex 96. In order to further stimulate viral gene expression, Tat recruits additional host factors including the human SEC (Fig. 4). SEC contains a set of factors implicated in regulation of pol II transcription elongation, and its core components include the scaffold proteins AFF1 or AFF4, P‐TEFb, elongation stimulatory factors ELL1 or ELL2, and transcription factors ENL and AF9 97, 98, 99, 100, 101. The function of the SEC is to stimulate elongation of transcription by increasing the processivity of pol II 98. A recent study established that the AFF1‐containing form of the SEC is more potent in supporting HIV‐1 transactivation than AFF4‐SEC 101. The ELL1/ELL2 components of the SEC promote Tat transactivation by suppressing pol II pausing and ELL2 is more effective than ELL1 in Tat transactivation 98.

The crystal structure of Tat‐P‐TEFb 102 and the recently published Tat‐AFF4‐P‐TEFb crystal structure 103 provide insights into the mechanism of Tat activation. Tat interacts with both cyclin T and CDK9 and induces conformational changes in P‐TEFb upon binding. These structural studies can help in designing small‐molecule compounds that disrupt the Tat‐P‐TEFb and the Tat‐AFF4 interactions to specifically inhibit HIV transcription without affecting normal P‐TEFb function. The central role that P‐TEFb plays in Tat‐dependent activation of latent HIV genomes makes CDK9 an attractive target for the development of novel HIV therapeutics.

### More P‐TEFb please for HTLV‐1 too

**Figure 5:**
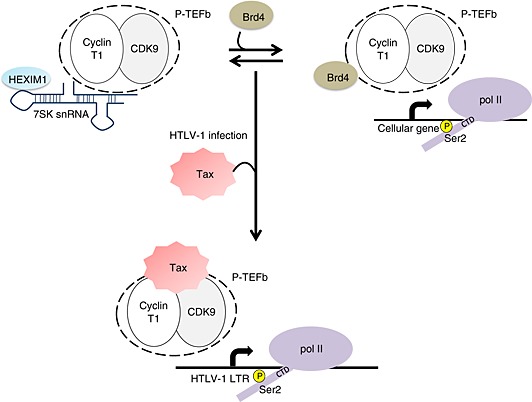
A dual function for Tax in modulating positive transcription elongation factor b (P‐TEFb) activity. Human T‐lymphotropic virus type 1 (HTLV‐1) transcriptional transactivator protein Tax disrupts the inactive 7SK snRNA/HEXIM1/P‐TEFb complex to create a novel Tax/P‐TEFb complex. Tax also acts as an antagonist of bromodomain‐containing the protein 4 (Brd4) and competes with Brd4 for interaction with P‐TEFb. The active Tax/P‐TEFb complex facilitates the phosphorylation of Ser2 of pol II carboxyl‐terminal domain (CTD) and enhances the activation of the HTLV‐1 long terminal repeat (LTR) promoter.

Human T‐lymphotropic virus type 1 (HTLV‐1) infections cause several human diseases such as adult T‐cell leukemia, the neurological disorder HTLV‐1‐associated myelopathy, and uveitis 104, 105, 106, 107. HTLV encodes a transcriptional transactivator protein called Tax, which functions as an activator of the long terminal repeat (LTR) promoter 108, 109. Similarly to HIV‐1 Tat, HTLV‐1 Tax influences transcription by pol II 110. P‐TEFb is essential for Tax transactivation *in vivo* and *in vitro*, and siRNA treatment to CDK9 and the CDK9 inhibitor flavopiridol both inhibit transactivation by Tax 110. *In vitro* binding studies demonstrate that Tax competes with Brd4 for P‐TEFb binding through direct interaction with cyclin T1. In addition, Tax overexpression decreases the amount of 7SK snRNA associated with P‐TEFb and specifically stimulates Ser2 phosphorylation of the pol II CTD 111. These events give rise to a functional Tax/P‐TEFb complex for viral LTR activation (Fig. 5). As Brd4 competes with Tax for P‐TEFb, LTR promoter activity is higher in HTLV‐1‐transformed Brd4‐deficient cells 112. Interestingly, a recent study showed that in common with HIV‐1 Tat transactivation, the ELL component of the pol II SEC is essential for Tax‐mediated transactivation. Tax enhances ELL incorporation into the histone acetyltransferase p300 and P‐TEFb transcriptional complexes. Depletion of ELL using an shRNA‐mediated approach abrogates Tax transactivation 113. Thus, the HTLV‐1 virus uses similar strategies to HIV‐1 to hijack cellular P‐TEFb.

### Inhibiting CDK9 blocks adenovirus replication

Human adenovirus (HAdV) is a double‐stranded DNA virus that causes upper and lower respiratory infections 114. The adenovirus large E1A (L‐E1A) protein is a transcriptional activator that functions through the action of an activation domain named “conserved region 3” (CR3). CR3 has been shown to recruit cellular transcription factors including mediator subunit 23 (Med23) 115, 116. Recent proteomic analysis in human cells infected with HAdv5 revealed that L‐E1A is associated with many subunits of the mammalian mediator complex 117. Interestingly, the presence of mediator subunits in the L‐E1A protein complex is greatly reduced in cells depleted of Med23, suggesting that Med23 links the transactivation domain CR3 with the mediator complex. The proteomic analysis also revealed that L‐E1A is associated with super elongation complex components, such as CDK9, cyclin T1, AFF4, ELL, and EAF. The catalytic constituent of the SEC, CDK9, was shown to be critical for the transcription of HAdv5 early genes by L‐E1A and HAdv5 replication 117. Interestingly, a newly developed CDK9 inhibitor, FIT‐039 (N‐[5‐fluoro‐2‐(1‐piperidinyl)phenyl]isonicotinthioamide), suppresses HAdV replication 118. This further suggests that HAdV hijacks P‐TEFb activity for viral‐specific transcription.

### P‐TEFb influences influenza

The best‐studied member of the orthomyxovirus RNA virus family is the influenza A virus, which is responsible for acute respiratory diseases in humans. After infection with influenza A, viral RNA‐dependent RNA polymerases (vRNPs) are transported to the nucleus, where viral transcription takes place. vRNPs associate with the promoter region of protein‐coding genes with lower levels in the regions downstream of the promoter 119. This reflects the distribution of pol II in infected cells. In mock‐infected and influenza virus‐infected cells, there are similar levels of pol II associated with the promoter region of genes. However, in infected cells, there are decreased levels of pol II in coding regions suggesting that influenza virus infection inhibits pol II elongation 120. Zhang *et al.* 121 demonstrated that CDK9/cyclin T1 interacts with the vRNP of influenza A virus and facilitates its association with cellular pol II phosphorylated on Ser2 of the CTD. CDK9 interacts with three subunits of influenza virus vRNP, PB1, PB2, and PA, when they are ectopically expressed in cells. Interestingly, in contrast to HIV transcription, the kinase activity of CDK9 is not crucial for influenza virus transcription as overexpression of the dominant negative form of CDK9 does not reduce influenza virus transcription activity. In addition, the CDK9 inhibitor DRB does not affect viral transcription 120. However, siRNA‐mediated knockdown of cyclin T1 inhibits viral mRNA synthesis and overexpression of cyclin T1 promotes vRNP activity. It was suggested that interaction with P‐TEFb facilitates vRNP association with cellular pol II for cap‐snatching 121. Thus, P‐TEFb, but not its kinase activity, plays an important role in regulating expression of the influenza A virus.

### P‐TEFb co‐operates with DENV‐C to activate an inflammatory response

Dengue virus (DENV) is a mosquito‐transmitted RNA virus and is one of the most common infectious pathogens worldwide 122. Viral infection results in dengue fever, dengue haemorrhagic fever, or dengue shock syndrome 123, 124. At present, an effective dengue vaccine or anti‐viral drug is not available. A notable issue that impedes dengue vaccine development is the limited understanding of the immunopathology of DENV infection. The DENV single‐stranded RNA molecule is about 11 kb in size. It encodes three structural proteins (core protein, non‐glycosylated membrane protein, and envelope protein) and seven non‐structural proteins 125. DENV infection is associated with increased levels of the inflammatory chemokines IL‐8, IFN‐γ, TNF‐α, and TNF‐β 126, 127, 128. A recent finding has shed new light of the role of P‐TEFb in the activation of IL‐8 gene expression during DENV infection 129. Li *et al.* 129 demonstrated that during DENV virus infection, P‐TEFb associates with DENV core protein (DENV‐C) to induce IL‐8 expression. DRB treatment or siRNA‐mediated knockdown of cyclin T1 prior to DENV infection abolishes the increase of IL‐8 induction. P‐TEFb and DENV‐C core protein co‐localize *in vivo* and co‐immunoprecipitate in DENV‐infected cells, and P‐TEFb and DENV core proteins are recruited to the IL‐8 gene. These findings further implicate the association of P‐TEFb with DENV‐C in altering host gene expression. The IL‐8 gene promoter contains sites for the transcriptional activator NF‐κB. Interestingly, in DENV‐infected cells, the DENV‐C protein appears to promote NF‐κB‐dependent activation only in the presence of P‐TEFb 129. The benefit to the virus of activating inflammation is unclear. However, the requirement of P‐TEFb association with DENV‐C to induce IL‐8 expression in DENV‐infected cells emphasizes the importance of P‐TEFb in DENV immunopathogenesis and suggests that P‐TEFb could be a valid drug target to treat some of the symptoms of DENV infection.

### CDK9 inhibitors as anti‐viral agents

The high mutation rate of viruses facilitates the emergence of strains resistant to drugs that target the virus directly, which represents a public health risk 130. In addition to combating viral drug resistance, the alternative strategy of targeting cellular proteins used by viruses increases the potential range of targets and could yield drugs active against several viruses. Given the importance of P‐TEFb for the replication of herpesviruses, HIV, HTLV, HAdV, influenza A virus and DENV, CDK9 and cyclin T are obvious potential drug targets. Drugs have already been developed that target P‐TEFb for the treatment of disease. For example, CDK9 inhibitors are currently in clinical trials for the treatment of human malignancies 131.

Given the regulation of CDK9 by viral proteins during HSV, EBV, HCMV, and KSHV infections, the potential use of CDK9 inhibitors as anti‐herpesvirus agents is particularly appealing. Acyclovir and its derivatives are effective, widely available, and have greatly reduced the burden of disease. However, the resistance to these drugs is becoming a major public health problem. The growing resistance is due to their frequent use in suppressive therapy and prophylaxis of HSV infection 132. There is therefore a pressing need for the identification of potential herpesvirus drug targets to facilitate the development of the next generation of anti‐herpesvirus drug. One of the most selective, non‐competitive inhibitors of CDK9 is flavopiridol 133. It was demonstrated that flavopiridol can suppress the replication of HSV, HCMV, HAdV, and HIV 88, 134. In HeLa cells, the anti‐HIV activity of CDK9 inhibitors, including flavopiridol, correlates with a dose‐dependent loss of the large form of P‐TEFb and reduction in HIV‐1 infectivity. Unfortunately, studies in primary cell cultures indicate that cytotoxicity of the drugs used is a major drawback 135. Interestingly, studies by Salerno *et al.* 136 demonstrated that effects of dominant negative CDK9 and flavopiridol are not equivalent. However, the next generation of small pharmacological compounds targeting CDK9 includes some promising anti‐HIV therapeutic agents.

Roscovitine, a purine‐derived CDK inhibitor, inhibits replication of HSV, HCMV, and HIV 137, 138, 139. Roscovitine also reduces tumor size and plasma EBV DNA in patients with nasopharyngeal carcinoma 140. Unfortunately, these inhibitors affect a wide range of CDKs and often negatively affect cell cycle progression 141. It was recently demonstrated that R22, a novel selective CDK9 inhibitor, possess anti‐cytomegaloviral activity 67. In addition, a new CDK9 inhibitor (FIT‐039) that exhibits an anti‐viral effect on DNA and RNA viruses was identified 118. It was shown to inhibit the replication of DNA viruses such as HSV‐1, HSV‐2, HAdV, and HCMV in cultured cells. Furthermore, FIT‐039 also suppresses the replication of influenza virus H1N1 and of HIV. Interestingly, FIT‐039 has a less significant effect on the cellular transcriptome than flavopiridol, and it does not affect cellular proliferation 118. It is therefore a prospective drug that affects a broad spectrum of viruses.

## Concluding remarks

P‐TEFb is an important regulator of cellular processes, and it is functionally integrated into the expression of many human viruses. In particular, the activity of CDK9 is crucial for the course of HSV, HCMV, EBV, HIV, HTLV, HAdV, DENV, and KSHV virus infection.

**Figure 6:**
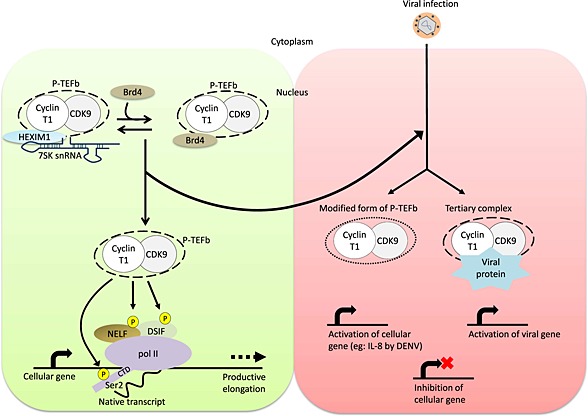
Positive transcription elongation factor b (P‐TEFb) as a master regulator of cellular and viral transcription. In uninfected cells (left panel), the negative elongation factor (NELF) and the DRB sensitivity‐inducing factor (DSIF) enhance pol II stalling. Subsequent recruitment of P‐TEFb allows phosphorylation of DSIF, NELF, and Ser2 of carboxyl‐terminal domain (CTD) of pol II, which leads to productive elongation. In the context of virus‐infected cells (right panel), various viral factors affect transcription by modulating or recruiting the activity of P‐TEFb. Consequently, the cellular genes are inhibited, whereas the viral genes become activated.

P‐TEFb is therefore a key player not only in cellular processes but also in viral biology (Fig. 6). Either by modifying its activity or regulating the amount of active and inactive complexes of P‐TEFb and/or physical interaction with components of P‐TEFb, viruses have evolved strategies to hijack this key factor via their own regulatory proteins and P‐TEFb functions as a central player in virus‐host interaction to facilitate the viral replication cycle. Importantly, studies of viruses that use P‐TEFb as a specific factor for efficient transcription will also help to elucidate the molecular mechanisms controlling transcription in uninfected cells.

## Supporting information

Supporting info item

Supporting info item

Supporting info item
