# Supplementary figures and images for: P‐TEFb goes viral
Source: Inside Cell. 2015 Nov 25;1(2):106–16. doi: 10.1002/icl3.1037 (PMC4863834; doi:10.1002/icl3.1037)

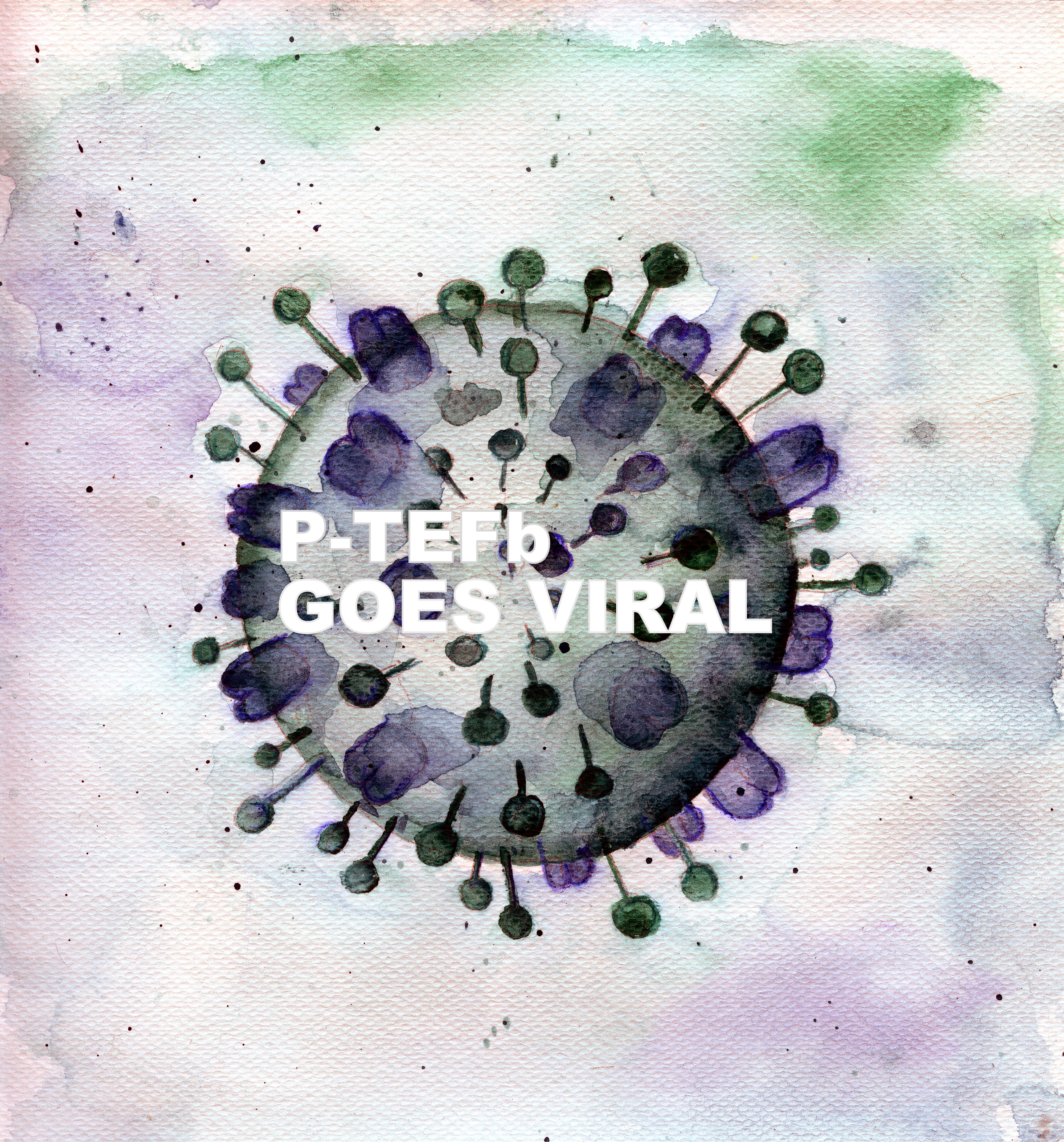

Supplement: Supplementary file 1 — Supporting info item [file ICL3-1-106-s001.jpg]

## Slide 1
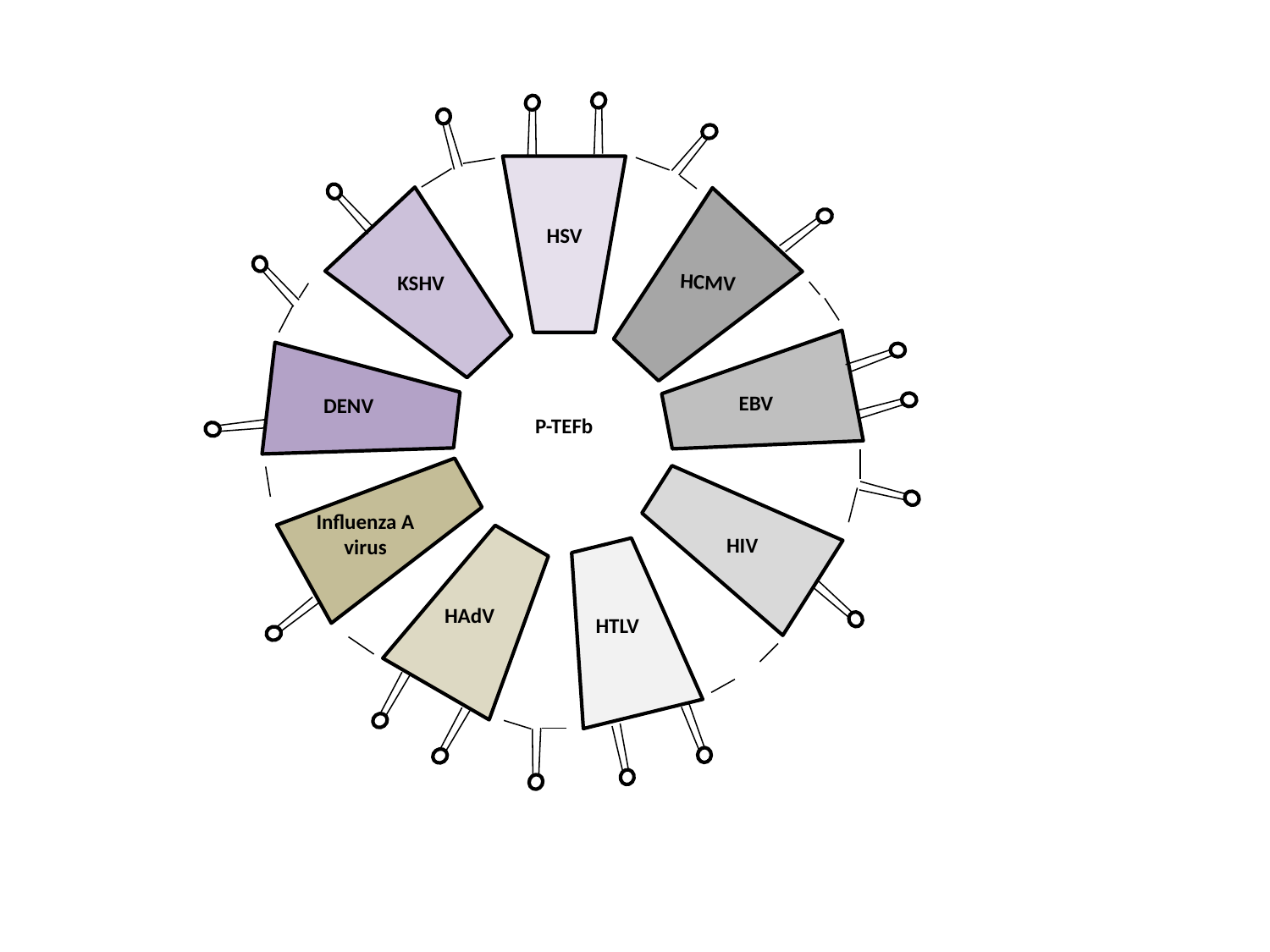

HSV
HCMV
KSHV
EBV
DENV
P-TEFb
Influenza A virus
HIV
HAdV
HTLV

Supplement: Supplementary file 2 — Supporting info item [file ICL3-1-106-s002.pptx]
