# Supplementary material for: P‐TEFb goes viral
Source: Inside Cell. 2015 Nov 25;1(2):106–16. doi: 10.1002/icl3.1037 (PMC4863834; doi:10.1002/icl3.1037)
Supplement: Supplementary file 3 — Supporting info item [file ICL3-1-106-s003.docx]

**Many viruses subvert P-TEFb function to facilitate viral gene expression.** P-TEFb is integral to the replication of a range of viruses, including, HSV, KSHV, CMV, EBV, HIV, HTLV, AdV, Influenza A and Dengue virus.
